# Evaluation of Calcium Carbonate Inhibitors Using Sintered Metal Filter in a Pressurized Dynamic System

**DOI:** 10.3390/ma12111849

**Published:** 2019-06-07

**Authors:** Adriana Velloso Alves de Souza, Francisca Rosário, João Cajaiba

**Affiliations:** 1 Instituto de Química, Pólo de Xistoquímica, Universidade Federal do Rio de Janeiro (UFRJ), Cidade Universitária, Rio de Janeiro 219410-614, Brazil; adrianavelloso@hotmail.com; 2 Centro de Pesquisas e Desenvolvimento Leopoldo Américo Miguez de Mello, PETROBRAS, Cidade Universitária, Rio de Janeiro 21941-915, Brazil; frosario@petrobras.com.br

**Keywords:** scale inhibitors, dynamic systems, calcium carbonate, adhesion, precipitation

## Abstract

Calcium carbonate scale is formed during oil and gas production. Tube-blocking tests (TBTs) are used to define the minimum inhibitory concentration (MIC) in order to prevent scale adhesion in the petroleum production system equipment. However, non-adhered crystals may favor heterogeneous nucleation to other deposits such as calcium naphthenates, causing a more severe scale problem, increasing production losses and treatment costs. The objective of the present work was to develop a new dynamic test methodology to determine the MIC for CaCO_3_ using a sintered metal filter. Organophosphorus inhibitors were selected for comparison with the conventional dynamic tube-blocking system. The results demonstrated that the use of the filter allowed an MIC of the inhibitors to be obtained considering the precipitation prevention. The inhibitor concentration in the conventional tube-blocking system does not prevent precipitation, acting only on adhesion and crystal growth on the capillary wall. Tests to evaluate the potential of calcium naphthenates formation in a naphthenate flow rig dynamic system demonstrated the influence of heterogeneous nucleation from non-adhered carbonate crystals, potentially aggravating deposition problems in oil and gas production systems.

## 1. Introduction

Calcium carbonate scale is one of the main flow assurance problems encountered during oilfield operations. This salt can agglomerate at different points in the production system, resulting in a partial or even total loss of production and additional operational costs for cleaning [1,2]. The general equilibrium involved in calcium carbonate precipitation [3,4] is defined as:

(1) $$Ca^{2+}\left(aq\right)+HCO_{3}^{-}\;\left(aq\right)+H_{2}O\;\left(l\right)\;⇌CaCO_{3}\;\left(s\right)+H_{3}O^{+}\left(aq\right)$$

Scale formation is a complex phenomenon involving thermodynamic supersaturation, kinetics, and hydrodynamic effects (surface shear stress and turbulence) [5]. Supersaturation is the boundary to trigger precipitation or crystallization processes. The saturation ratio (SR) of a solution is defined as:

(2) $${\mathrm{SR}}^{2}=a{\mathrm{Ca}}^{2+}\;{{\mathrm{CO}}_{3}}^{2-}/kps_{CaCO_{3}}$$

where a represents activity of the ions in solution (Ca^2+^ and CO_3_^2−^) [4,6]. When SR > 1, the solution is supersaturated, and the precipitation process is spontaneous. The Multiscale® software can be used to calculate SR as well as mass balance for Ca^2+^ and CO_2_ in the aqueous phase to each temperature, pressure, and water composition.

The application of chemical inhibitors is an alternative technology to prevent or minimize scale formation in many industrial processes [7,8]. The scale inhibitors work by preventing either precipitation and/or adherence of the scale at threshold (sub-stoichiometric) concentrations [9]. 

Inhibitor performance, in terms of MIC or the threshold concentration required to prevent scale (calcium carbonate) is the most important aspect for scale control additives [9]. The laboratory tests used in the industry are based upon performance tests for inhibition of both static bottle and dynamic tube-blocking types. Both test procedures are commonly adopted to that described in the NACE standard TM 0374-2016 [10] and NACE standard TM 31105-2005 [11], respectively. The static bottle test evaluates the effectiveness of scale inhibitors in the prevention of bulk homogeneous precipitation. The bottle tests suffer a number of significant limitations when examining calcium carbonate formation and its chemical treatment. The determination of the MIC depends on the quantification of the ions in solution using techniques such as inductively coupled plasma optical emission spectrometry (ICP-OES) or atomic absorption spectrometry [12]. These techniques require a higher quantity of consumables, calibrations, and specialized technical knowledge. The bottle tests do not have the ability to overcome the natural buffering capacity of the carbonic acid/bicarbonate/CO_2_ equilibrium (which tends to increase the in situ pH) due to non-renewal of fluids [13]. When carried out in a stove, bottle tests have limitations of ambient pressure and temperature below 100 °C, which may impact on the results applied in the field. Therefore, the bottle tests can only be used as a pre-screening tool. However, for calcium carbonate scale, the impact of pressure, temperature (including dissolution at lower temperatures), and pH control makes such routine bottle testing significantly less appropriate, mainly under HPHT (high pressure and high temperature) conditions. In such cases, pressurized systems are more suitable for examining carbonate scale in order to ensure in situ pH. It will be possible to control the temperature and pressure in the flow conditions representative of the oil field. Other studies [13,14,15,16] described the impact of pressure and pH on the carbonate scale condition.

The dynamic conventional TBTs evaluate the effectiveness of scale inhibitors on preventing the heterogeneous nucleation and growth at metal surfaces (scale adhesion) in a capillary tube under dynamic flow conditions [9,17,18,19]. This ability gives dynamic TBTs an advantage over static jar tests in that scale growth and inhibitor performance can be measured readily at temperatures higher than 100 °C (maximum up to 200 °C) and also at elevated pressures (typically up to 5000 psi). The system back pressure presence also reduces the potential for CO_2_ released from the aqueous solution and the consequential impact this would have on system pH and carbonate scaling potential. The dynamic tests, therefore, allow scale formation and inhibition to be examined under conditions more closely resembling those encountered in oilfield production [13]. However, the flow in a laminar system does not reproduce the turbulent conditions of the oilfield pipelines and lines. References [20,21,22,23,24] using electrochemical, quartz crystal microbalance and/or rotating cylinder electrode (RCE) techniques have also been reported in the literature to evaluate inhibitors on growth and change of CaCO_3_ crystal morphology as well as the influence of coating of the metallic surface. These techniques estimate the scale deposition directly on a metallic surface, but they have a limited range of temperature (60 °C) and atmospheric pressure [12]. Previous literature presents the sintered metal filter blocking system to evaluate the efficiency of inhibitors and parameters that influence the formation of barium sulfate and calcium naphthenates. Schalge and Dormish [25] evaluated the inhibitor’s ability to prevent the formation of barium sulfate in a dynamic flow system consisting of a scaling coil and in-line filter. However, the blocking system uses the filter only to collect the solid although the pressure transducers continuously monitor the differential pressure in the scaling coil and filter. The authors observed crystals accumulated in the scaling coil outlet without any increase in pressure recorded. Therefore, the use of an in-line scaling coil and filter may be problematic for the efficiency of the scale inhibitors’ evaluation. This system allows growth and adhesion of crystals in the capillary, and non-adhered crystals are retained in the filter. However, if the fouling potential is high, the capillary may be blocked before any crystals are retained in the filter. The inhibitor will only evaluate adhesion efficiency, not precipitation. For dynamic systems where only the filter is used, the inhibitor may act to inhibit precipitation and prevent the formation of fouling crystals. Nichols et al. [26] and other works [1,27] demonstrated the development of dynamic flow equipment termed a naphthenate flow rig, using a sintered metal filter to evaluate the parameters (i.e., temperature, pH, calcium concentration, bicarbonate and CO_2_) favoring the formation of calcium naphthenate and its stable emulsions as well as the performance of different commercial inhibitors of calcium naphthenates. These studies used conventional dynamic tube blocking equipment, under the same parameters employed in the naphthenate flow platform, to determine a suitable dose of carbonate inhibitor. However, the results showed that the carbonate crystals were still observed inside the walls of the mixing cell and potentiated the naphthenates formation. Because conditions promoting calcium naphthenate formation (pressure drop causing CO_2_ loss by increasing pH) are similar to those that cause calcium carbonate scale, mixed deposits often occur [26]. Therefore, it is recognized that test protocols with a prediction of mixed deposits require an accurate evaluation of the inhibition of carbonate precipitation.

This work describes the use of a novel methodology, using a sintered metal filter to conduct carbonate precipitation experiments under pH, temperature, and pressure conditions more similar to field conditions. The advantages of these proposed methods in relation to the conventional tube blocking (laminar flow) are in the regime of turbulent flow generated by the passage of fluids through the filter pores, and the prediction of the onset potential of carbonate precipitation, mainly in scenarios where suspended particles may remain in the bulk fluid and be transported to surface facilities, causing co-precipitation with calcium naphthenates. Although the pore size of a sintered metal filter has been specified, the results obtained and reported in this paper are relatively generic, and therefore, can be applied to other pore size filters for even more severe scaling environments.

## 2. Materials and Methods 

### 2.1. Materials

**Figure 1:**
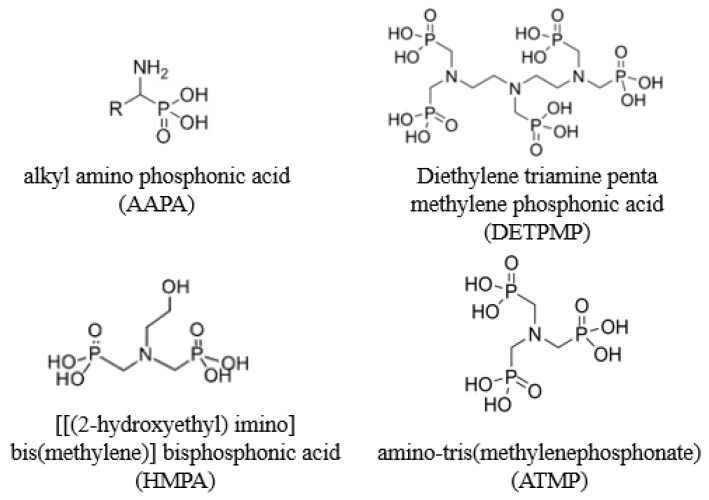
Generic structures of scale inhibitor organophosphorus used in dynamic systems.

The sintered metal filter and the scaling coil (loop) used in the dynamics systems were 316L stainless steel provided by the company Swagelok. Chemical performance tests were undertaken using commercial scale inhibitors organophosphorus. The inhibitors were diethylene triamine penta methylene phosphonic acid (DETPMP), alkyl amino phosphonic acid (AAPA), (((2-hydroxyethyl) imino) bis(methylene)) bisphosphonic acid (HMPA), and amino-tris(methylenephosphonate) (ATMP). Barium chloride dihydrate, calcium chloride dihydrate, sodium bicarbonate, sodium chloride, strontium chloride hexahydrate, hydrochloric acid (37%), sodium hydroxide microbeads, and n-heptane P.A. were all analytical grade supplied by Vetec Química Fina Ltda, Duque de Caxias, Brazil. Naphthenic acids extract was prepared from calcium naphthenates deposit field samples, hydrochloric acid, and n-heptane. Figure 1 presents a description of the generic inhibitors tested in this work. 

### 2.2. Synthetic Water Preparation

The synthetic brines represent produced water from a field with a similar ionic composition, salinity, and pH. These synthetic brines are prepared in the laboratory by dissolving the appropriate inorganic salts in deionized water. The composition of the synthetic formation brine used in this study represents a field brine composition from a Brazilian petroleum reservoir presenting calcium carbonate precipitation potential [26]. The brine composition is given in Table 1. This synthetic brine was divided into two solutions, defined as cation brine and anion brine. These solutions were prepared separately by weighing the appropriate quantity of salts and mixing with deionized water, and then mixed at a ratio of 1:1 during experiment runs. Cation and anion brines were filtered through 0.45-µm membrane filter paper. The appropriate quantity of concentrated aqueous NaOH and HCl solution was added immediately prior to each test in order to produce a pH of 6.4.

### 2.3. Determination of Scale Inhibitor MIC for Calcium Carbonate by Dynamic System in Filter or Scaling Coil

**Figure 2:**
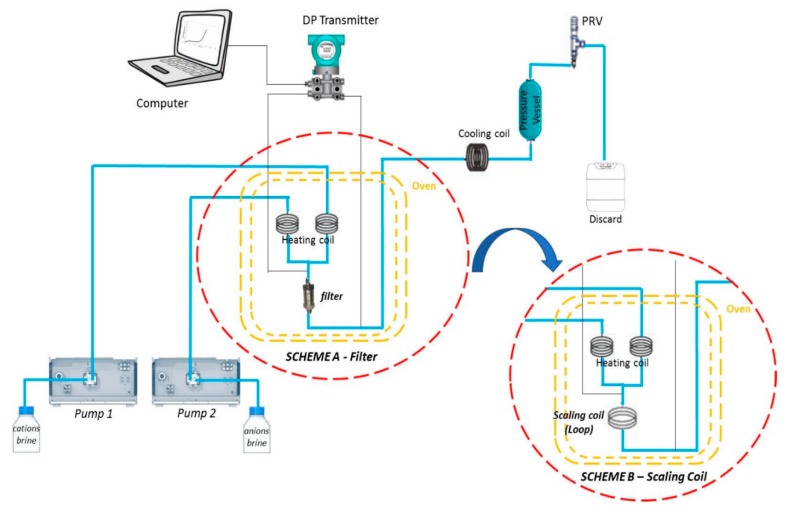
Schematic of dynamic scale system: Scheme A—using a filter with 7 µm pores and Scheme B—using a scaling coil with 500 µm inner diameter.

The dynamic systems presented in this paper were developed by Scaled Solutions Ltd. (Livingston, UK). The schemes are shown in Figure 2. The dynamic tests for calcium carbonate precipitation in a 7 µm filter (Scheme A) and conventional dynamic tests in scaling coil (Scheme B) with a 500 µm inner diameter (referred to as a TBT) were performed.

**Figure 3:**
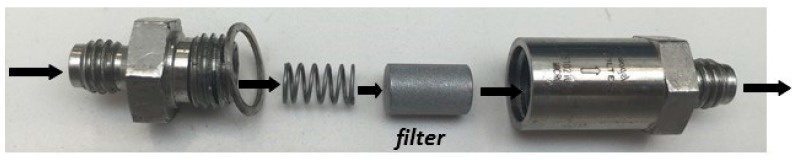
Schematic of turbulent flow in the dynamic system using a 316 stainless steel filter.

In this test, the brine is separated into two aqueous fluids, one was termed cation brine (containing the cationic ions: sodium, calcium, barium, and strontium as chloride) and the other anion brine (containing the anionic ions: sodium chloride and sodium bicarbonate). The cations and anions brines were injected by pumps 1 and 2, respectively, and percolated in separate capillaries, conditioned at 80 °C and 44 psi. These two brines were pumped at flow rates (5.0 mL/min each) that define the mixing ratio between them (in this case, 50:50). Scale inhibitors were injected in the anions’ brine for MIC determination. The fluids were mixed, and the differential pressure increases in the 7-µm pore filter (Scheme A) or scaling coil with 1 m length and 0.5 mm inner diameter (Scheme B) were determined. A summary of dynamic test conditions is presented in Table 2. The increased differential pressure indicates that blockage was occurring, which, in turn, was indicative of calcium carbonate scale. The filter was replaced with each test, and the system was cleaned with an acid solution, containing 5.0% acetic acid and 7.0% formic acid and with deionized water. A schematic of the turbulent flow in the dynamic system using a filter is shown in Figure 3. 

### 2.4. Deposition of Calcium Naphthenate by Dynamic Filter System

The dynamic calcium naphthenate evaluation was performed on a naphthenate flow rig. A schematic of the dynamic system is available in Figure S1 in the Supplementary Materials. The organic fluid was composed of a heptane extract containing naphthenic acids extracted from a field deposit extract, and the brine containing scale inhibitor ATMP was then added. The procedure for extraction of the naphthenic acids used here was adapted from Bertelli et al. [28]. The fluids were conditioned at a temperature of 80 °C and pressure of 44 psi in the oven. The fluids were mixed in a static mixer and a mixing valve to produce tight in situ emulsions similar to those which may occur during normal production conditions. The fluids then pass through two in-line filters (coarse filter/baffle and a fine 7 µm filter) to a separator (sapphire cell). The differential pressure was monitored across a fine filter throughout the test and it provided an indication of blockages occurring, which, in turn, was indicative of calcium naphthenate solid formation. In addition, the separating cell was transparent, enabling the emulsion formation in the separator to be observed.

## 3. Results

### 3.1. Dynamic Tests

**Figure 4:**
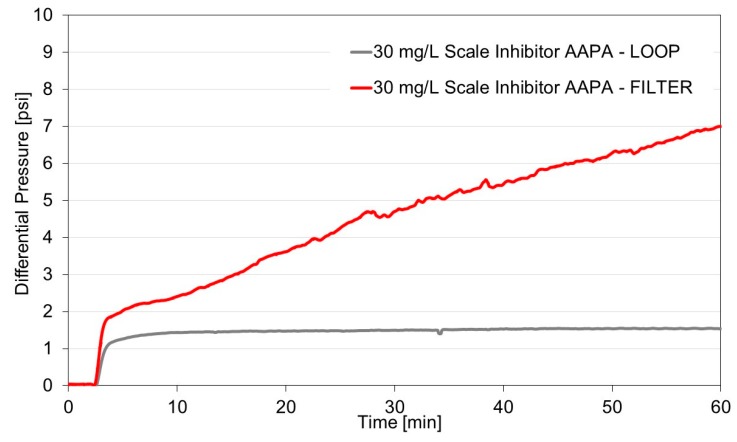
Carbonate precipitation in the filter (FILTER) and scaling coil (LOOP) dynamic systems at 80 °C and 44 psi, Inhibitor AAPA (30 mg/L).

The conventional TBT used to evaluate the effectiveness of calcium carbonate inhibitor in the dynamic system was performed using a scaling coil and is standardized using the NACE 31105 method [10]. The increase of the differential pressure at the inlet and outlet of the capillary indicates a blockage occurrence due to the carbonate crystal adhesion to the wall of the capillary (also termed a loop). An efficient inhibitor should have an MIC capable of preventing adhesion and growth of scaling in the capillary. As shown in Figure 4, the MIC determined in the loop for the AAPA inhibitor, for example, was 30 mg/L (gray curve—LOOP). As can be seen, there is no change in the differential pressure during one hour of testing. However, this test only refers to the efficiency of the inhibitor on adhesion.

When the capillary was replaced by a porous filter, restricting the size of the crystals formed, and not only adhered, it was observed (red line—FILTER) that carbonate precipitation occurred and its crystals were retained in the filter, causing a differential elevation of pressure. As can be seen, 30 mg/L of inhibitor did not prevent carbonate precipitation. As crystals larger than the filter pores form, they are retained in the filter and the differential pressure increases, indicating that precipitation has occurred. A higher concentration of inhibitor is then required to prevent clogging of the filter pores. Therefore, at a concentration of 30 mg/L, the AAPA inhibitor is able to prevent adhesion, but cannot prevent carbonate precipitation.

**Figure 5:**
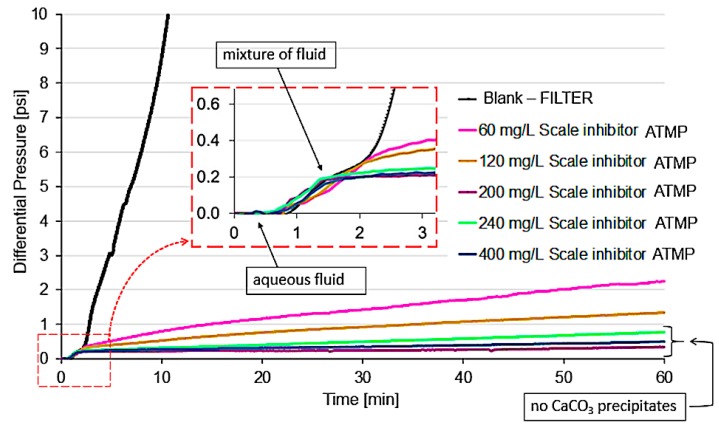
Scale inhibitor ATMP concentrations (0 mg/L, blank test; 60 mg/L; 120 mg/L; 200 mg/L; 240 mg/L; and 400 mg/L) used for MIC determination in a filter dynamic system at 80 °C and 44 psi.

The organophosphorus inhibitors were evaluated to determine the MIC for calcium carbonate precipitation in the dynamic filter system. In Figure 5, the differential pressure measurement graph is shown, as obtained for the dynamic efficiency experiment of calcium carbonate inhibition in a filter with amino-tris(methylenephosphonate) (ATMP) inhibitor addition. As brine containing calcium ions (cation brine) and brine containing bicarbonate ions (anion brine) come together in the filter, the first calcium carbonate crystals are formed, and crystals larger than the pore diameter are retained, causing an increase in differential pressure until complete filter saturation occurs. The black line represents the experiment in the absence of a scale inhibitor (blank test). The filter saturation occurred at around 10 min (blank test), demonstrating a high potential for calcium carbonate scale, a result which was expected according to the saturation ratio (SR) of the calculated synthetic production water. The composition of this synthetic formation water, similar to the formation water composition associated with an oil field in Brazil, indicated an SR for carbonate precipitation equal to 88 in the condition of pH 6.4, 80 °C, and 44 psi, representing a high risk of precipitation.

**Figure 6:**
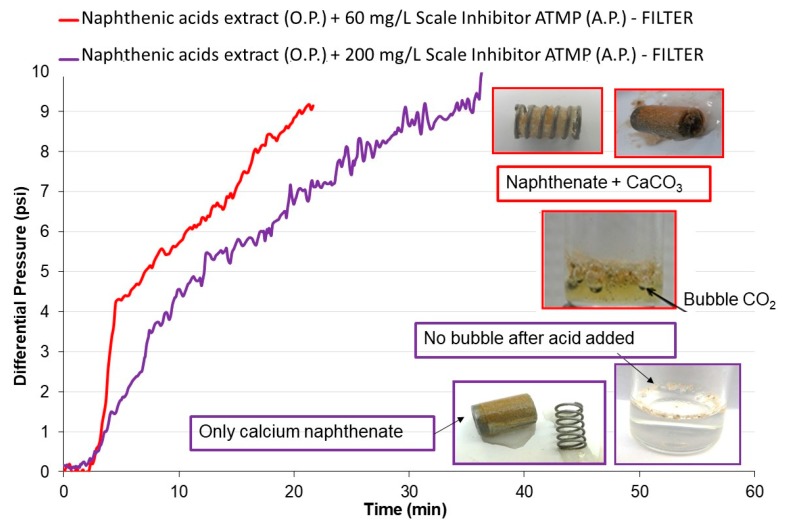
Dynamic efficiency of 7 µm filter scale (naphthenate flow rig) using naphthenic acid extract or pure heptane solvent in the organic phase with addition of scale inhibitor ATMP in anions brine at 80 °C and 44 psi. O.P.—organic phase; A.P.—aqueous phase.

In the graph, the differential pressure variation was corrected by the initial distilled water flow and the load loss caused by the filter porosity. Therefore, all experiments presented here started with zero differential pressure. Moreover, the differential pressure increase obtained by the fluid mixture and solution salinity was not considered as the onset of scale, but determined the baseline after the signal stabilization, as highlighted in Figure 5. Thus, only an increase of 1 psi in the differential pressure from 0.2 psi (baseline value after mixing) was considered as the onset of scale. This baseline value may change depending on the viscosity of the inhibitor added to the test. A maximum time of 1 h was established for all tests in the presence of scale inhibitor. These parameters of time and differential pressure are in agreement with other studies available in the literature [29,30]. When a certain inhibitor concentration acts to prevent a differential pressure variation higher than 1 psi in 1 h of testing, crystals larger than the filter pores are not formed, and so, that concentration is the MIC or efficiency. In Figure 6, the results of dynamic tests for the ATMP inhibitor are presented, which showed efficiency to the inhibition of the precipitation of calcium carbonate when used at the MIC of 200 mg/L. The differential pressure measurements for the comparison between the concentrations of inhibitor evaluated present random uncertainties inherent to the system of less than 1 psi; thus, they do not influence MIC determination. Using the same methodology, others organophosphorus inhibitors (diethylene triamine penta methylene phosphonic acid (DETPMP), alkyl amino phosphonic acid (AAPA), (((2-hydroxyethyl) imino) bis(methylene)) bisphosphonic acid (HMPA), and amino-tris(methylenephosphonate) (ATMP)) were evaluated. The N/P ratio and MIC of inhibitors for calcium carbonate by using a conventional TBT dynamic system and dynamic sintered metal filter system at 80 °C and 44 psi are shown in Table 3.

The calcium carbonate inhibition efficiency is provided by MIC determination, comparing different inhibitors. It can be observed in Table 3 that the organophosphorus inhibitor AAPA demonstrated an MIC of 30 mg/L, and DETPMP, HMPA, and ATMP inhibitors showed the same MIC (60 mg/L) in the scaling coil. This result indicates that in the capillary from the presence of two phosphonic groups, there was no difference in inhibitor concentration, and the same relation is reflected for N/P ratios below 0.60. However, an increased MIC of these inhibitors was observed when the filter was used because of the need to prevent carbonate precipitation that blocks the filter pores. MICs of 90, 100, 100, and 200 mg/L were obtained for AAPA, DETPMP, HMPA, and ATMP inhibitors, respectively, in the filter system. The differential pressure measurement graph for DETPMP, AAPA, HMPA, and ATMP inhibitors in the dynamic systems are available, respectively, in Figures S2–S5 in the Supplementary Materials. In both systems, the AAPA inhibitor presented better inhibition efficiency compared to the MIC values of the other inhibitors evaluated. The differential pressure showed a variation of less than 1 psi, initially indicating that even though some crystals had formed, there was no adhesion to the scaling coil walls, and that no filter pore obstruction due to calcium carbonate precipitation in the dynamic filter system occurred. 

### 3.2. Case Study

To exemplify this phenomenon of secondary nucleation of calcium naphthenates from the presence of carbonate crystals, a test using the naphthenate flow rig system was performed to evaluate the potential for deposition of calcium naphthenates. This system also used a porous filter to detect the naphthenate deposition and formation of stable emulsions in Figure 6, the tests are shown, as performed with naphthenic acid extract and brine containing 60 and 200 mg/L of ATMP inhibitor, respectively, for red and lilac curves.

It can be seen in Figure 6 that initially only the aqueous fluid passes, followed by the mixture with the organic fluid (extract). An increase in the filter differential pressure was observed only by mixing the fluids and the emulsion formation to approximately 4.0 psi and 2.5 psi, respectively, for the curves with 60 and 200 mg/L of ATMP inhibitor. From this value, any elevation is considered deposition by calcium naphthenates, which occurs shortly thereafter, before 10 min of testing for the two curves. However, by removing the carrier containing a spring and the 7 µm filter (see Figure 6), the presence of carbonate precipitate was observed, even with the addition of 60 mg/L of the inhibitor. The highlighting of the spring and red filter in Figure 6 illustrates that the carbonate was confirmed by the release of CO_2_ when adding a solution of hydrochloric acid in the tank collected in the filter. It is worth remembering that the addition of acid to the calcium naphthenate deposit would only cause protonation of the carboxylate to form naphthenic acids. On 200 mg/L inhibitor concentration was observed that the differential pressure increase occurred only as a function of naphthenates deposition, which was confirmed by the absence of bubbles (CO_2_ release) when hydrochloric acid was added to the collected precipitate in the spring and purple filter, highlighted in Figure 6. 

The calcium carbonate presence enhanced naphthenate deposition, and the MIC determined in the conventional dynamic system (60 mg/L) was not sufficient to prevent carbonate precipitation. Therefore, in a real production system, this result would be devastating and would cause significant operational and financial losses. On the other hand, the MIC (200 mg/L) carbonate determined in the filter dynamic system allowed only the deposition of naphthenates to be evaluated in the naphthenate flow rig system.

## 4. Discussion

### 4.1. Dynamic Tests

In conventional dynamic TBTs, the phenomena of supersaturation, precipitation, and adhesion occur in the scaling coil also referred to as a loop, with a shorter contact time (around 10 s) in these laminar flow systems. According to Goodwin et al. [5], in standard TBT dynamic tests, scale is formed under laminar flow and shear conditions. However, these conditions are not representative of the turbulent high shear fluxes that are frequently encountered during oil and gas production. The conventional TBT reproduces only the adhesion on metallic surfaces occurring in the reservoir pipes, differently from the porous filter dynamic system, which allows the detection of the inhibition efficiency of the carbonate precipitation rather than just detecting the adhesion of the scale on metallic surfaces, a subsequent process to carbonate precipitation. Therefore, the tendency is for smaller crystals to be formed at a higher proportion and carried by the constant flow in the scaling coil, initially not causing variation in differential pressure. However, the adhesion of these small nuclei to the scaling coil wall allows crystal growth, and hence initiation of the inlay. Then, the MIC determined in the scaling coil represents only the concentration on which the inhibitor acts in the deposition of calcium carbonate crystals on a field pipe wall. 

This filter type allows a surface filtration to occur. The carbonate crystals are retained on the filter element surface, forming a surface of solids until the filter element is partially or totally closed, reducing the efficiency of filtering or saturating. This saturation can be monitored by the differential pressure at the inlet and outlet of the filter. Therefore, this porous filter has the capacity to retain particles that are larger than its pores, which were 7 µm in this work.

Venâncio et al. [6] evaluated the impact of monoethyleneglycol (MEG) on calcium carbonate scale by monitoring particle size distribution and found an increase in the fine particle count, corresponding to a size smaller than 10 μm in the precipitation experiments of calcium carbonate without MEG. A count between 50 and 150 μm was attributed to the evaluation of the carbonate crystals’ growth. Cao et al. [31] studied the effects of nucleation on micro-calcium carbonate (1 µm–1 mm) in processes of hydration. They observed that carbonate crystals with 0.7 or 3 μm size provide additional nucleation sites for the formation and development of other solids. The sintered filter, selected for this work, was composed of trapped fine particles in a dense matrix. The nominal pore size was 7 μm with a range in pore size of 5–10 μm. The filter can remove 95% of particles larger than the nominal pore size [32]. This filter is commercially available and can be considered to evaluate the calcium carbonate precipitation. This order of magnitude is approximately 70 times higher than the internal diameter of the scaling coil (500 μm) used in conventional TBTs.

When inhibitor is present in the brine, it may act on the inhibition mechanism of the carbonate nucleation and/or crystal growth depending on inhibitor concentration. The mechanism of the filters towards the calcium carbonate inhibition, using the dynamic filter system, is based on crystal growth inhibition and crystal size exclusion. The inhibitor concentration efficiency, determined by the MIC, acts on the crystallization process to avoid crystals formation larger than the filter pores, which in this work was 7 µm. Tighter protocols may use filters with pore sizes smaller than 7 µm to further restrict the nuclei size formed; however, a higher inhibitor dosage will be required. In capillaries, the inhibitor concentration required to retard capillary blockage is lower. The absence of a porous filter prevents the crystals’ size from being restricted, rendering the particles more prone to growth in the aqueous phase dispersion, resulting in adhesion on the capillary wall.

**Figure 7:**
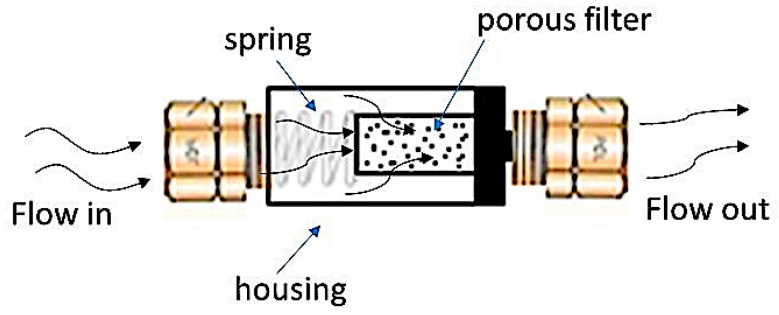
Illustration of the turbulent flow in the dynamic system using the porous filter.

In the filter, the levels of shear stress and turbulence were increased as a function of the barrier caused by the presence of the filter, where its pores cause a variation in the speed and direction of the flow (see Figure 7), and consequently results in increased potential for scale formation. Qualitatively, this system responds in a manner which is more similar to the effect of shear and turbulence occurring in the field.

In high-temperature systems, calcium carbonate precipitation becomes more critical because the solubility of this salt decreases with increasing temperature. Therefore, higher dosages of inhibitors are then expected to be required in both systems (filter and capillary) for calcium carbonate inhibition.

**Figure 8:**
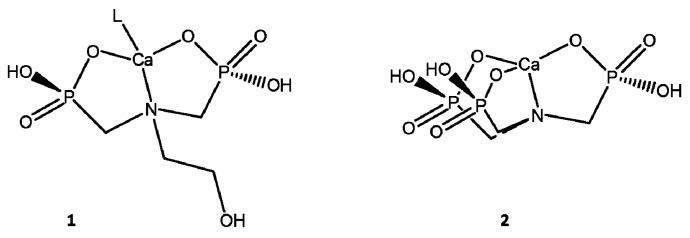
Chelate rings formed when (1) HMPA inhibitor and (2) ATMP inhibitor were 50% dissociated.

Structurally, the difference between the inhibitors is in the number of phosphonic groups, and only due to the replacement of a phosphonic group by a hydroxymethylene group with the HMPA inhibitor. It is known that the higher number of phosphonic groups in the molecule, the better their inhibition efficiency. However, it is yet to be confirmed because that the AAPA inhibitor has only one phosphonic group and obtained the best inhibition efficiency among the inhibitors. According to Shaw et al. [33] considering the rings formed in the reaction with calcium, 5–6 atom rings (including Ca^2+^) are more thermodynamically stable than larger rings; therefore, the higher the number of these rings the chelate forms, the higher the inhibition efficiency. Rings of 5–6 atoms formed for the HMPA and ATMP inhibitors when their protons were 50% dissociated are shown in Figure 8. 

The ATMP inhibitor has more phosphonic groups and forms chelates with three rings of five atoms, whereas the HMPA inhibitor forms only two, thus, it would be expected that the HMPA inhibitor would have increased inhibition efficiency. However, this observation is contrary to the results obtained by Shaw et al. [33]. A possible explanation for the result obtained here is in the relationship between nitrogen and phosphorus in each molecule of inhibitor. The relationship is shown in Table 3, and it can be observed that as the N/P ratio decreases in the inhibitor molecules, the inhibition efficiency increased.

Carbonate crystals not adhered can act as a secondary nucleus to potentiate other types of deposition, as in the case of calcium naphthenates. Therefore, in fields where there is a risk of mixed deposition of naphthenates and carbonates, the determination of the MIC through dynamic filter systems can prevent this type of nucleation, but the same prevention cannot be obtained with the MIC determined in the conventional tube-blocking system.

### 4.2. Case Study

Calcium naphthenates form as micelles through interaction of the carboxylate (deprotonated naphthenic acid) with two calcium ions in one single step. Because calcium ions are not likely to penetrate the hydrophobic core of the micelle, only reactions of calcium ions with external carboxylate groups of the molecule proceed. In the petroleum field, calcium naphthenate deposition can be aggravated by the presence of inorganic scale such as calcium carbonate. Carbon crystals function as seeds to induce naphthenate deposition. The heterogeneous nucleation reduces the energy necessary for the formation of an initial surface for deposition growth. This phenomenon is better observed when there is an increased contact time between fluids containing precipitating ions (RCOO^−^ and Ca^2+^) and solids (CaCO_3_) suspended and/or adhered to a surface. The naphthenic acids interact with the calcium retained in the crystalline carbonate by modifying the initial carbonate structure.

The dynamic calcium carbonate inhibition efficiency tests using a porous filter are able to detect carbonate precipitation, rather than only assessing the scale adhesion on the capillary surface, according to conventional dynamic systems. The results obtained can reduce costs associated with remediation of organic and inorganic depositions incorporated into the crystalline network of calcium carbonate.

## 5. Conclusions

The use of a sintered metal filter allowed the presence of calcium carbonate crystals in suspension to be evaluated, making the determination of the inhibition efficiency of carbonate precipitation possible. This approach is important in terms of considering the impact of the presence of calcium carbonate crystals on the aggravation of other types of fouling in the oil and gas production system, as in the case of calcium naphthenates. In TBTs, the MIC is only effective for adhering the crystals to metal surfaces, not considering flow solids. Therefore, a methodology that allows the inhibition of carbonate precipitation is more advantageous in terms of mixed-scale remediation costs than a methodology based only on adherence to metal surfaces.

## Figures and Tables

**Table 1:** Brine composition.

Ion	Concentration/(mg/L)
Sodium	69,529
Calcium	18,000
Barium	300
Strontium	1700
Chloride	140,000
Bicarbonate	1000

**Table 2:** Summary of dynamic tests conditions.

SCHEME A		SCHEME B	
Sintered Metal Filter		Scaling Coil (Conventional Tube Blocking Test—TBT)	
Flow	Turbulent	Flow	Laminar
Filter dimensions (diameter × height)	7.67 mm × 12.65 mm	Scaling coil length	1 m (1000 mm)
Filter nominal pore size Filter pore size range	7 µm 5–10 µm	Scaling coil ID	0.5 mm (500 µm)
Filter metallurgy	316 stainless steel	Scaling coil metallurgy	316 stainless steel
Combined flow rate	10 mL/min	Combined flow rate	10 mL/min
Run test duration	1 h	Run test duration	1 h
Failure criteria	Increase in ΔP ≥ 1 psi	Failure criteria	Increase in ΔP ≥ 1 psi
Temperature	80 °C	Temperature	80 °C
Pressure	44 psi	Pressure	44 psi

**Table 3:** MIC for calcium carbonate using conventional TBT dynamic system and dynamic sintered metal filter system at 80 °C and 44 psi.

Inhibitor	N/P Ratio	MIC (mg/L) in TBT Dynamic System	MIC (mg/L) in Dynamic Filter System
AAPA	1.0	30	90
DETPMP	0.60	60	100
HMPA	0.50	60	100
ATMP	0.33	60	200

## Supplementary Materials

The following are available online at https://www.mdpi.com/1996-1944/12/11/1849/s1, Figure S1: Schematic of naphthenate flow rig, Figure S2: Determination of MIC of calcium carbonate to scale inhibitor DETPMP in dynamic systems at 80 °C and 44 psi: (a) in loop, (b) in filter, Figure S3: Determination of MIC of calcium carbonate to scale inhibitor AAPA in dynamic systems at 80 °C and 44 psi: (a) in loop, (b) in filter, Figure S4: Determination of MIC of calcium carbonate to scale inhibitor HMPA in dynamic systems at 80 °C and 44 psi: (a) in loop, (b) in filter, Figure S5: Determination of MIC of calcium carbonate to scale inhibitor ATMP in dynamic systems at 80 °C and 44 psi: (a) in loop, (b) in filter.
