# Supplementary material for: Evaluation of Calcium Carbonate Inhibitors Using Sintered Metal Filter in a Pressurized Dynamic System
Source: Materials (Basel). 2019 Jun 7;12(11):1849. doi: 10.3390/ma12111849 (PMC6600948; doi:10.3390/ma12111849)
Supplement: Supplementary file 1 [file materials-12-01849-s001.pdf]

materials

Supplementary Materials

Evaluation of Calcium Carbonate Inhibitors Using Sintered Metal Filter in a Pressurized Dynamic System

Adriana Velloso Alves de Souza [1], Francisca Rosário [2] and João Cajaiba [1,*]

[1] Instituto de Química, Pólo de Xistoquímica, Universidade Federal do Rio de Janeiro (UFRJ), Cidade Universitária, Rio de Janeiro 219410-614, Brazil; adrianavelloso@hotmail.com
[2] Centro de Pesquisas e Desenvolvimento Leopoldo Américo Miguez de Mello, PETROBRAS, Cidade Universitária, Rio de Janeiro 21941-915, Brazil; frosario@petrobras.com.br
* Correspondence: cajaiba@iq.ufrj.br

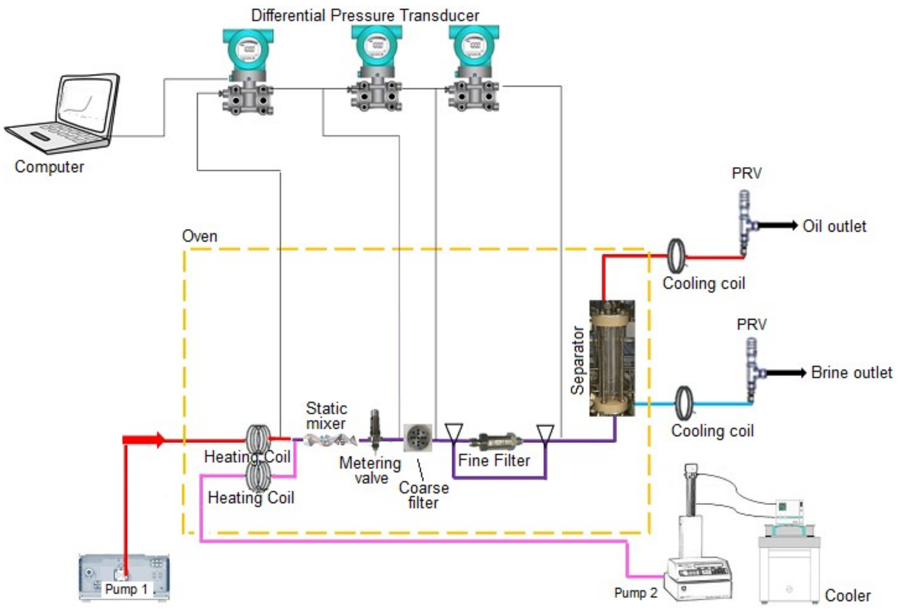

Figure S1. Schematic of the naphthenate flow rig.

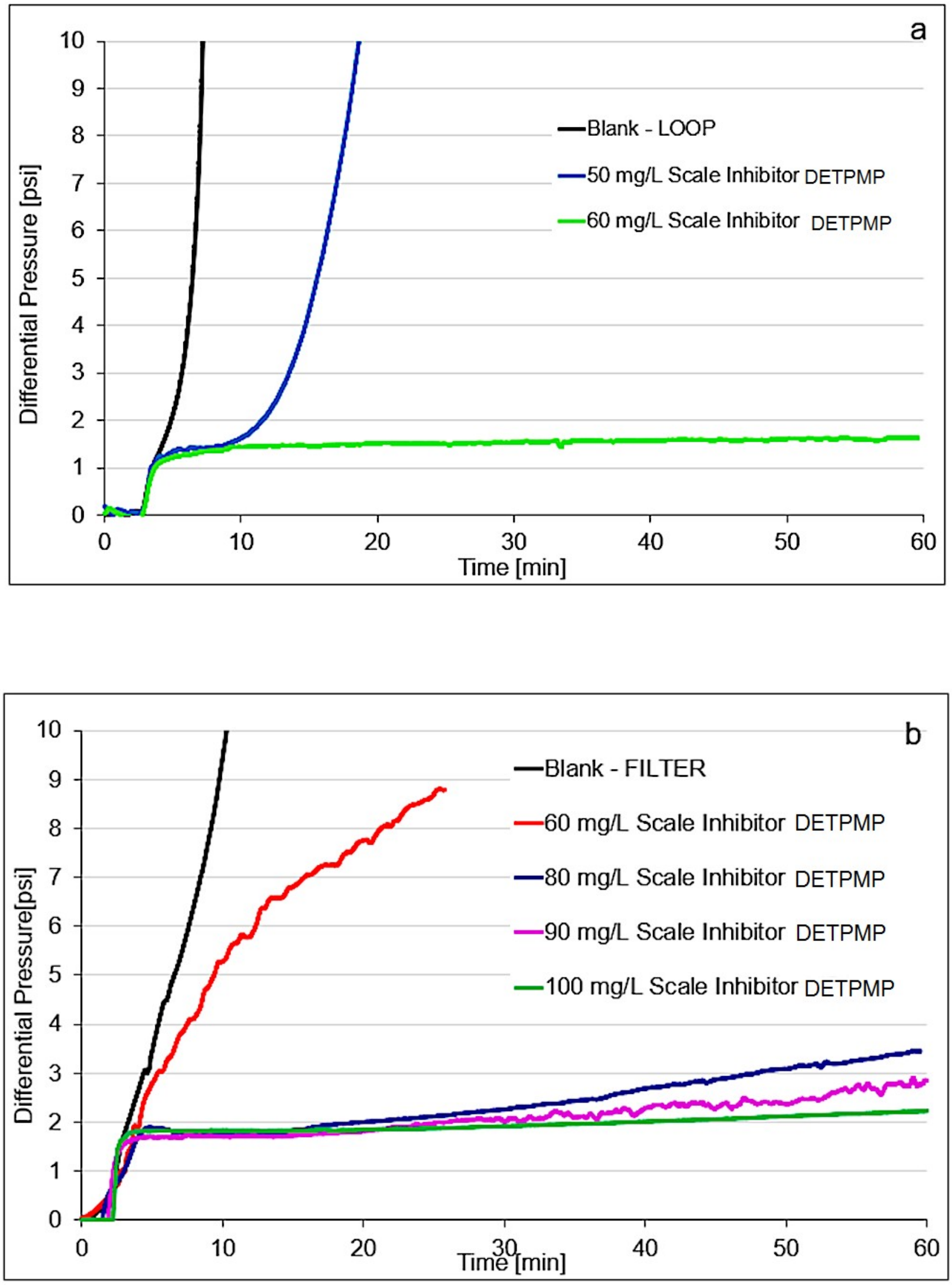

Figure S2. Determination of MIC of calcium carbonate to scale inhibitor **DETPMP** in dynamic systems at 80 °C and 44 psi: (a) in loop, (b) in filter.

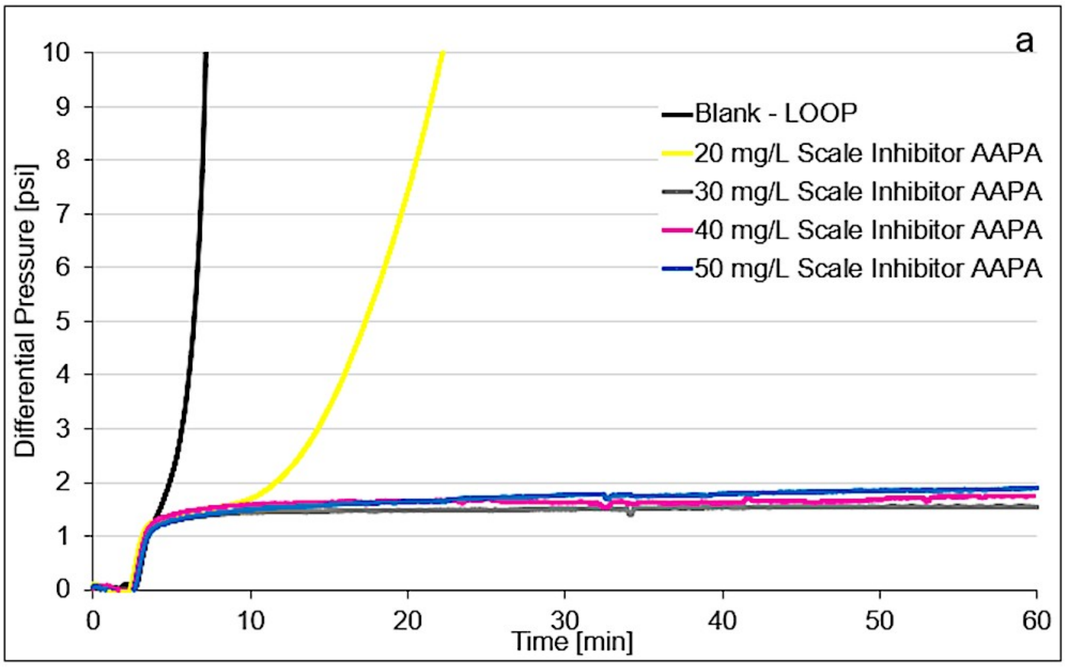

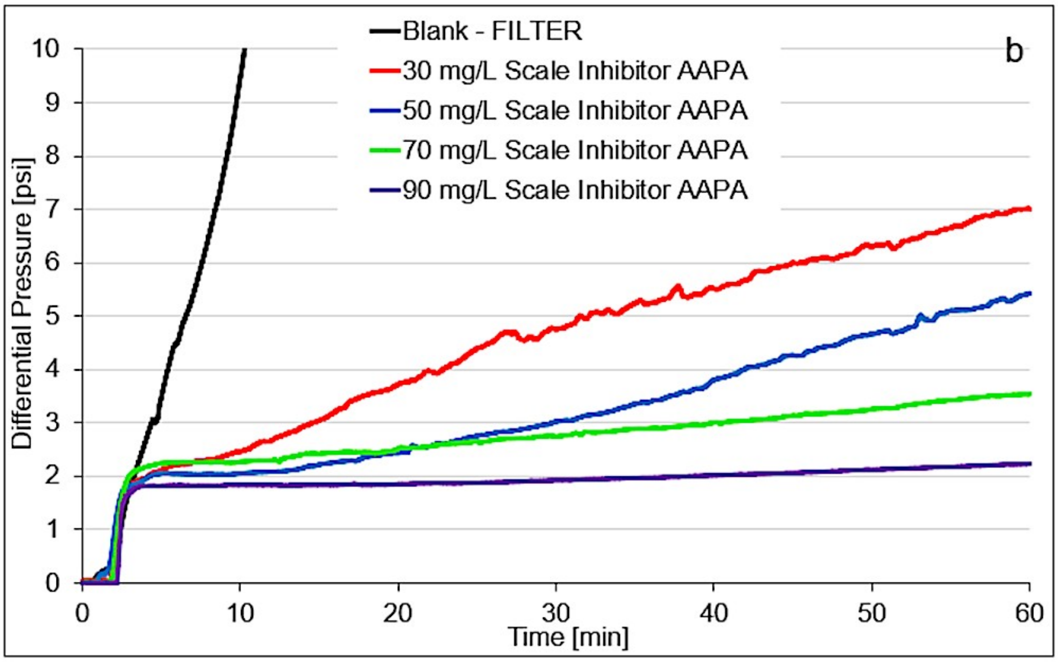

Figure S3. Determination of MIC of calcium carbonate to scale inhibitor **AAPA** in dynamic systems at 80 °C and 44 psi: (a) in loop, (b) in filter.

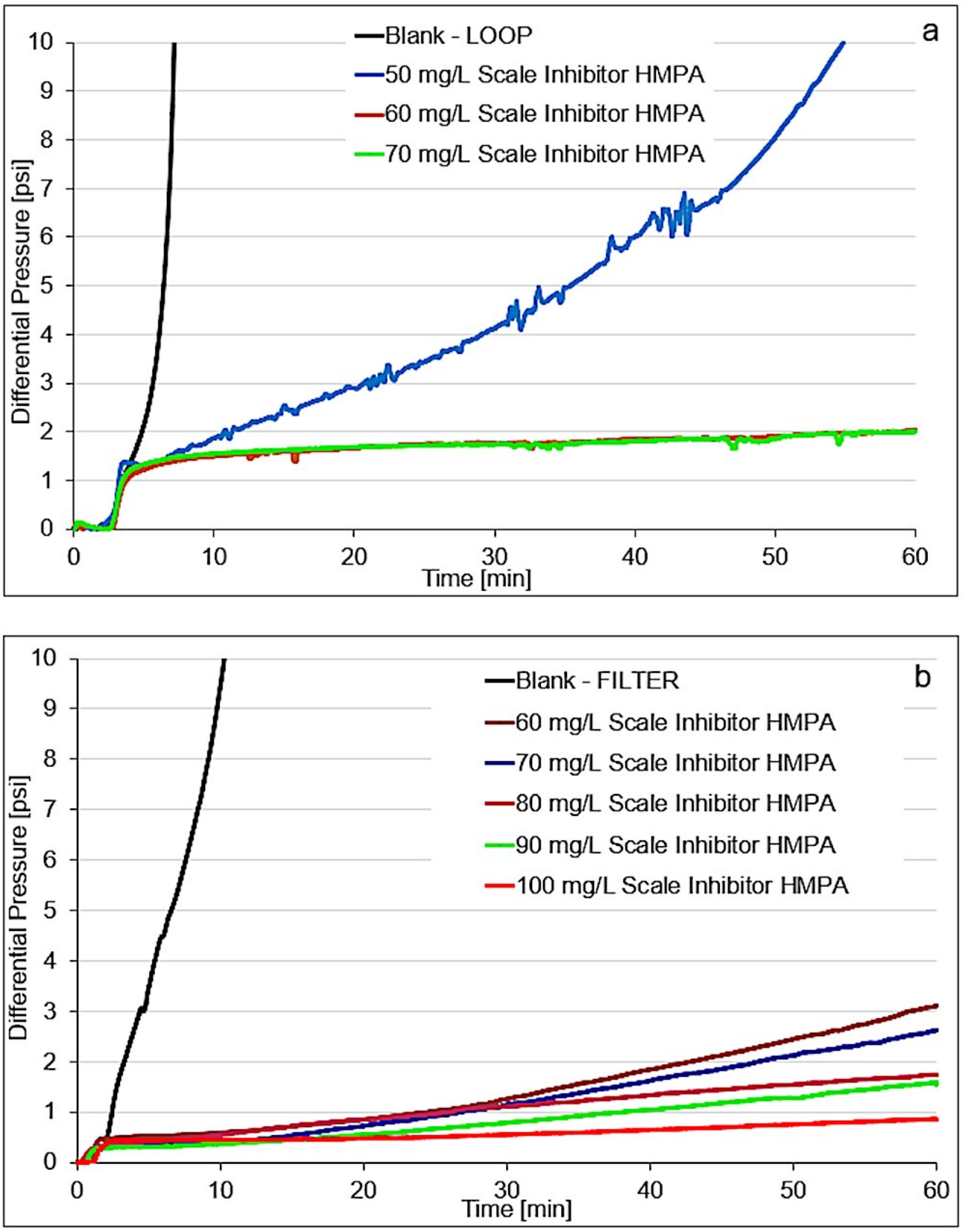

Figure S4. Determination of MIC of calcium carbonate to scale inhibitor **HMPA** in dynamic systems at 80 °C and 44 psi: (a) in loop, (b) in filter.

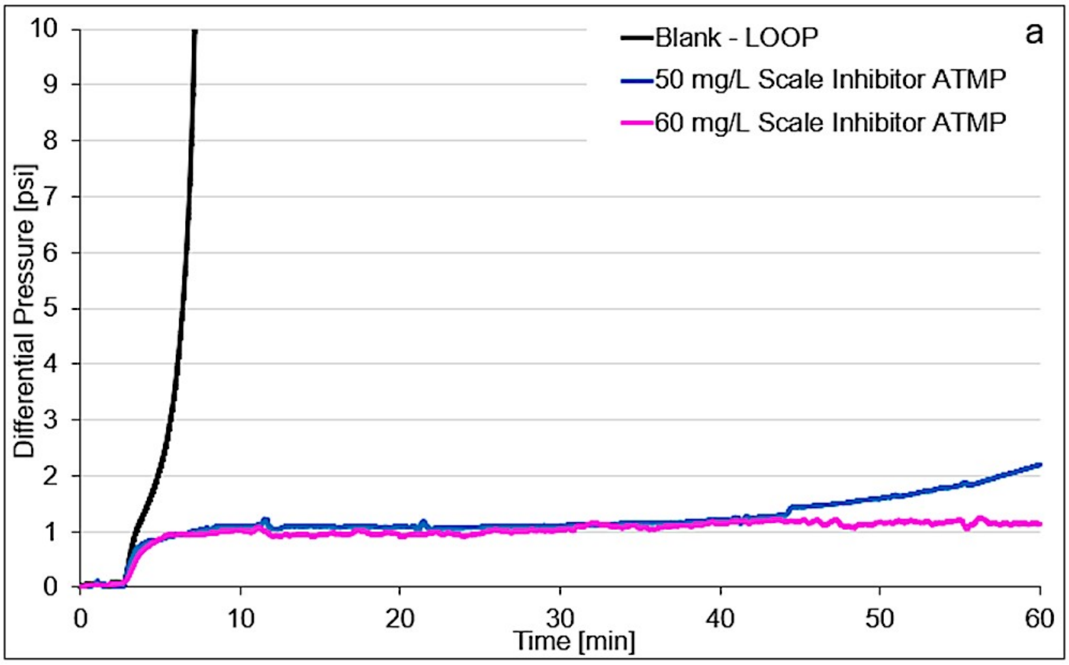

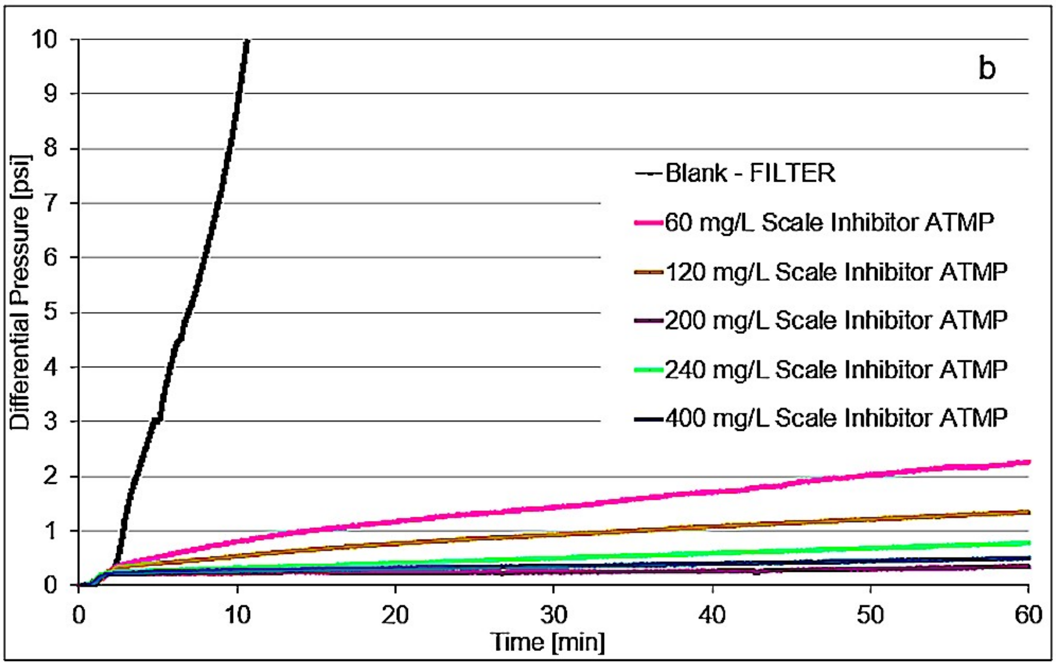

Figure S5. Determination of MIC of calcium carbonate to scale inhibitor **ATMP** in dynamic systems at 80 °C and 44 psi: (a) in loop, (b) in filter.

© 2019 by the authors. Submitted for possible open access publication under the terms and conditions of the Creative Commons Attribution (CC BY) license (http://creativecommons.org/licenses/by/4.0/).
